# Natural history of eosinophilic esophagitis: a systematic review of epidemiology and disease course

**DOI:** 10.1093/dote/doy015

**Published:** 2018-03-31

**Authors:** N J Shaheen, V Mukkada, C S Eichinger, H Schofield, L Todorova, G W Falk

**Affiliations:** 1 Center for Esophageal Diseases and Swallowing, University of North Carolina School of Medicine, Chapel Hill, North Carolina; 2 Division of Gastroenterology, Hepatology and Nutrition, Cincinnati Children's Hospital Medical Center, University of Cincinnati College of Medicine, Cincinnati, Ohio; 3 PharmaGenesis London, London, UK; 4 Shire, Zug, Switzerland; 5 Division of Gastroenterology, Hospital of the University of Pennsylvania, University of Pennsylvania Perelman School of Medicine, Philadelphia, Pennsylvania, USA

**Keywords:** eosinophilic esophagitis, esophagitis, esophagus, gastroenterology, gastrointestinal disease

## Abstract

Eosinophilic esophagitis is a chronic immune-mediated esophageal disorder. For its timely diagnosis, clinicians must recognize common symptoms, and understand differences in symptoms across patient groups. The aim of this study is to systematically review the epidemiology and natural history of eosinophilic esophagitis. The MEDLINE, Embase, and Cochrane databases were searched from 1974 to February 2017 for studies describing the epidemiology and natural history of eosinophilic esophagitis. Congress abstracts from 2014 to 2016 were also searched. Search results were screened against predetermined inclusion/exclusion criteria by two independent reviewers, and data extraction was performed in accordance with the Preferred Reporting Items for Systematic Reviews and Meta-Analyses guidelines. Of 1376 articles identified, 47 met the inclusion criteria: 20 on epidemiology and 27 on natural history. Incidence and prevalence of eosinophilic esophagitis varied widely across North America and Europe, and increased over time. Incidence increased 131-fold in the Netherlands (1996–2010), 20-fold in Denmark (1997–2006), and 5.1-fold in Calgary, Canada (2004–2008). The most commonly reported symptoms were emesis and abdominal pain in children, and dysphagia and food impaction in adults. Age at diagnosis was 5.9–12.0 years in children, and approximately 30 years in adults. Time between symptom onset and diagnosis was 1.2–3.5 years in children and 3.0–8.0 years in adults. Diagnostic delay was associated with an increased risk of endoscopic features of fibrostenosis. Symptoms of eosinophilic esophagitis differed significantly by age and race. In conclusion, there is an increasing incidence and prevalence of eosinophilic esophagitis. The considerable delay between symptom onset and diagnosis suggests that clinicians do not readily recognize the disease, which may have important clinical ramifications.

## INTRODUCTION

Eosinophilic esophagitis (EoE) is a chronic, immune-mediated disorder of the esophagus, which is associated with a large number of eosinophils infiltrating the esophageal mucosa.^1^ The predominant symptoms in EoE are dysphagia, feeding dysfunction, food impaction, chest pain, gastroesophageal reflux disease (GERD)-like symptoms, abdominal pain, vomiting, and anorexia, with symptom presentation varying with the age of the patient.^1^

EoE was described as a distinct disease entity more than two decades ago, and consensus recommendations on its diagnosis were published for the first time in 2007 and updated in 2011 and 2017.^1–3^ Given improvements in diagnostic techniques and increasing awareness of the disease, a thorough understanding of the evolving epidemiology of EoE is essential. Furthermore, common symptom patterns and disease progression of EoE are incompletely described.

This systematic review addresses two objectives. First, it evaluates the incidence and prevalence of EoE in children and adults, including trends over time. Second, it elucidates the symptom patterns and the natural history of EoE in all age groups.

## MATERIALS AND METHODS

### Identification of studies

The systematic review was designed to identify literature that included data on the epidemiology and natural history of EoE in children and adults.

Searches were carried out using search strategies including a combination of free text and Medical Subject Headings (MeSH) terms (Supplementary Table S1). Epidemiology searches queried EoE and search terms related to incidence, prevalence, risk factors, and trend analysis. Natural history searches queried EoE and search terms related to disease features, functional abilities (e.g. physical, cognitive, and psychiatric), severity and frequency of signs and symptoms, time course and predictors of manifestation progression, and survival.

Searches were conducted in Ovid^®^ on February 27, 2017 using: MEDLINE^®^ In-process & Other Nonindexed Citations and Ovid MEDLINE (covering publications from 1946 to present), Embase^®^ (covering publications from 1974 to present), and Evidence-Based Medicine Reviews, comprising the Cochrane Database of Systematic Reviews, the American College of Physicians Journal Club archives, the Database of Abstracts of Reviews of Effects, the Cochrane Central Register of Controlled Trials, the Cochrane Methodology Register, the Health Technology Assessment Database and the National Health Service Economic Evaluation Database. Searches were limited to studies in humans and English language publications. Supplementary searching included manual review of abstracts from congresses held from 2014 to 2016 by the following organizations: the American College of Gastroenterology; the North American Society for Pediatric Gastroenterology, Hepatology and Nutrition; the American Academy of Allergy, Asthma & Immunology; the American College of Allergy, Asthma & Immunology; the International Society for Pharmacoeconomics and Outcomes Research Annual International Meeting; and Digestive Diseases Week. Further publications were identified on the basis of assessment of the reference titles listed in articles selected for inclusion in this study and in systematic reviews identified in the searches described above.

### Study selection

Once publications had been identified, they were independently screened and evaluated by two independent reviewers based on their title and abstract in accordance with the 2009 Preferred Reporting Items for Systematic Reviews and Meta-Analyses (PRISMA) guidelines.^4^ Disagreements between reviewers regarding study selection were resolved by consensus.

To be included in the full-text review, publications had to meet specific criteria defined in a predetermined protocol (Supplementary Table S2). Searches included studies of adults and/or children with EoE. In agreement with the most recent European consensus guidelines,^3^ studies were included regardless of whether they excluded individuals with symptoms of GERD or patients who were responsive to proton pump inhibitor (PPI) treatment. Natural history searches excluded interventional studies. Studies were restricted to English language publications.

### Data extraction

Data extraction was carried out by one researcher using a standardized data extraction sheet, and these extractions were reviewed and confirmed by a second independent researcher. A third researcher performed a quality check of all extracted data. Data were presented as reported in the identified publications without applying any meta-analytic techniques.

The extracted data from epidemiology studies included the name of the first author, year of publication, study population, time period of estimate, sample size, diagnostic and exclusion criteria, and annual prevalence and incidence values per 100 000 people. Data ranges indicating the lowest and highest annual prevalence or incidence values reported in a given time period of estimate were also extracted.

Data extracted from natural history studies included the name of the first author, year of publication, study region, ethnicity, gender and age of study participants, diagnostic criteria, and information on whether patients with GERD or proton pump inhibitor-responsive esophageal eosinophilia (PPI-REE) were excluded in the study. Moreover, the four most common symptoms reported in each study were summarized in a table and reported both as absolute numbers and as the proportion of patients in a study population (in percentages). We further assessed the mean age and mean duration of symptoms at diagnosis (in years; including standard deviations, if available) and analyzed symptoms by age category as proportion of patients (in percentages).

### Assessment of quality of evidence

The identified studies were evaluated for quality of evidence using the Newcastle–Ottawa scale for quality assessment of cohort, case–control and cross-sectional studies (Supplementary Table S3).

## RESULTS

### Identified papers

**Fig. 1:**
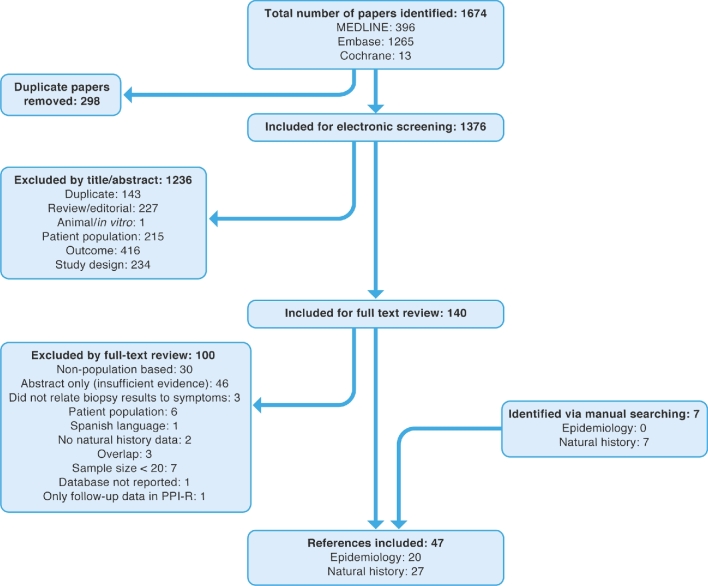
PRISMA diagram of included and excluded studies. An initial systematic review covered the existing literature published until February 1, 2016. An update to the systematic review was carried out covering literature published from January 1, 2016 to February 27, 2017 (the searches were designed to overlap by 1 month to allow for indexing lag within the databases). The identified references from both searches are combined in this PRISMA diagram. PPI-R, proton pump inhibitor-resistant; PRISMA, Preferred Reporting Items for Systematic Reviews and Meta-Analyses.

The searches identified 1674 articles, of which 1376 were screened after removal of duplicates. In total, 47 references met the inclusion criteria (Fig. 1); of these, 20 articles reported on the epidemiology and 27 on the natural history of EoE.

### Assessment of the epidemiology of EoE

#### Characteristics of epidemiology studies

**Fig. 2:**
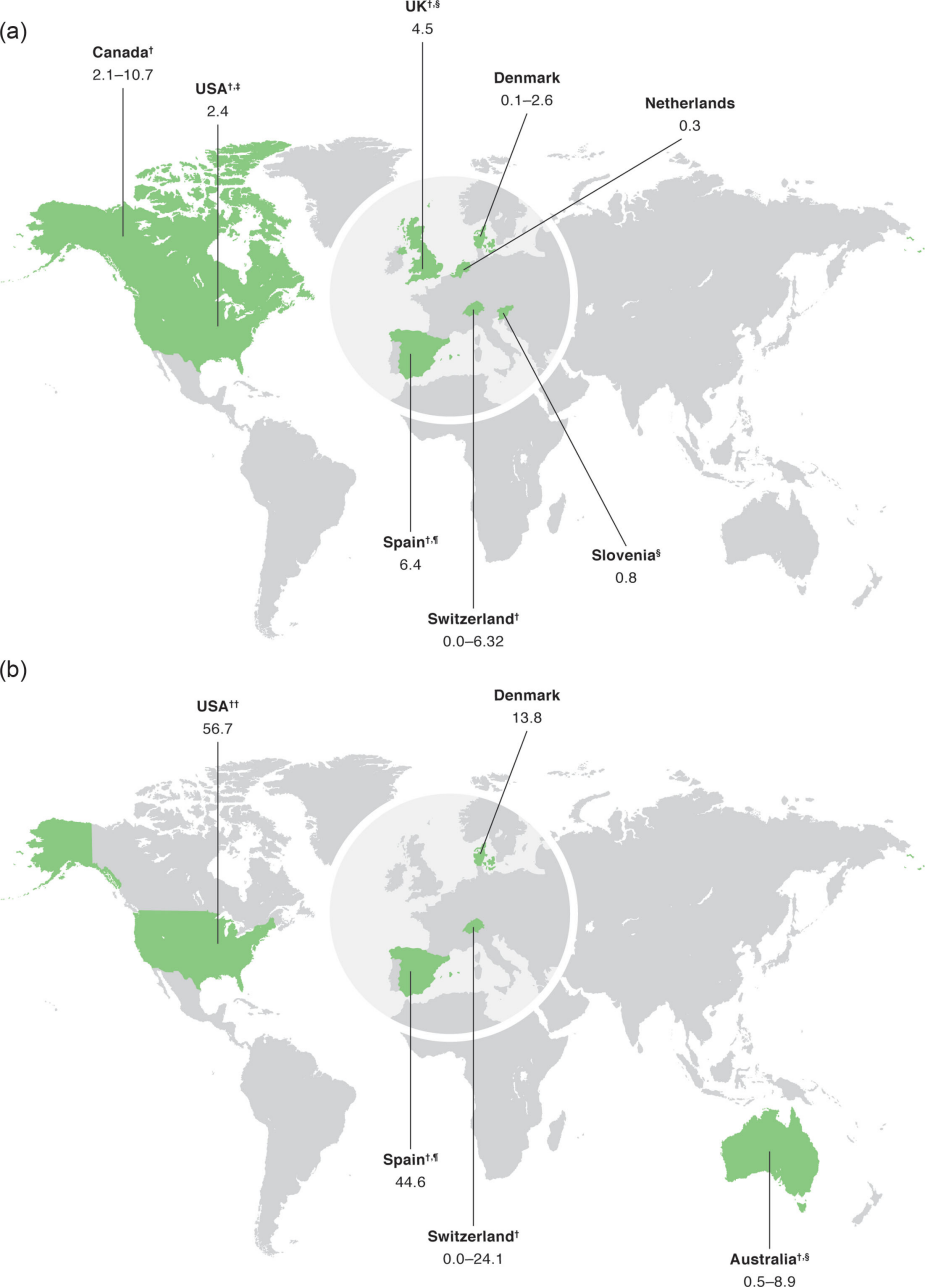
(a) Incidence and (b) prevalence of EoE. Representative data are presented for each country as incidence per 100 000/year and prevalence per 100 000. For each country where data are available, rates were taken either from the only study available (even if it relates only to a region) or from the study with the largest sample size. ^†^Data relate to a region of this country. ^‡^Age- and sex-adjusted values are reported. ^§^Study population included children only. ^¶^Study population included adults only. ^††^For the USA, we represented data from Dellon *et al*.^6^ instead of Mansoor & Cooper^13^ owing to the use of more standard diagnostic criteria. EoE, eosinophilic esophagitis; NR, not reported; PY, person-years.

Data on the incidence and prevalence of EoE and trends over time were gathered from 20 studies conducted in North America (Canada and USA),^5–13^ Europe (Denmark,^14,15^ Ireland,^16^ Netherlands,^17^ Slovenia,^18^ Spain,^19^ Switzerland^20–22^ and UK^23^), and Australia^24^ (Fig. 2 and Supplementary Table S4).

#### Incidence and prevalence

Incidence and prevalence estimates varied widely across the USA, Canada, Europe, and Australia (Fig. 2 and Supplementary Table S4). Studies also varied widely regarding methods of identifying cases of EoE; three relied on ICD-9 codes,^5,6,8^ one used the SNOMED-CT diagnosis,^13^ one used a combination of SNOMED and ICD-10 codes,^14^ and 15 used histology alone for the diagnosis of EoE.^7,9–12,15–24^ Of the studies using histology for the diagnosis of EoE, the majority used a cut-off of 15 or more eosinophils per high-power field.^7,10–12,14–20,23^

In the USA, prevalence estimates in children ranged from 7.3 per 100 000 per year in 1995–2004 in West Virginia,^7^ to 50.5 per 100 000 across the USA for 2009–2011.^6^ In adults, prevalence estimates across the USA ranged from 9.5 per 100 000 in 2008–2009^5^ to 58.9 per 100 000 in 2009–2011.^6^ The incidence of EoE in adults and children in North America ranged from 0.35 cases per 100 000 person-years in 1991–1995 in Minnesota, USA,^10^ to 10.7 cases per year in 2008 in Calgary, Canada.^11,12^ In European studies, no cases of EoE were identified at all in 1993–2003 in the Canton of Vaud in Switzerland,^20^ while the prevalence of EoE in adults and children was reported to be 42.8 per 100 000 in Olten County, Switzerland, in 2007–2009.^21,22^

The largest study analyzed the prevalence of EoE in the USA in 2010–2015 using the Explorys database, covering a source population of 30 301 440 patients.^13^ The overall period prevalence was 25.9 per 100 000. The prevalence of EoE was significantly higher in male patients than in female patients (odds ratio [OR] 2.00, 95% confidence interval [CI] 1.92–2.10). It was also significantly higher in Caucasians than in Asians and African-Americans (OR 2.00, 95% CI 1.86–2.14), and in the adult population (18–65 years of age) compared with children or the elderly (OR 1.63, 95% CI 1.54–1.71).

#### Trends over time

**Fig. 3:**
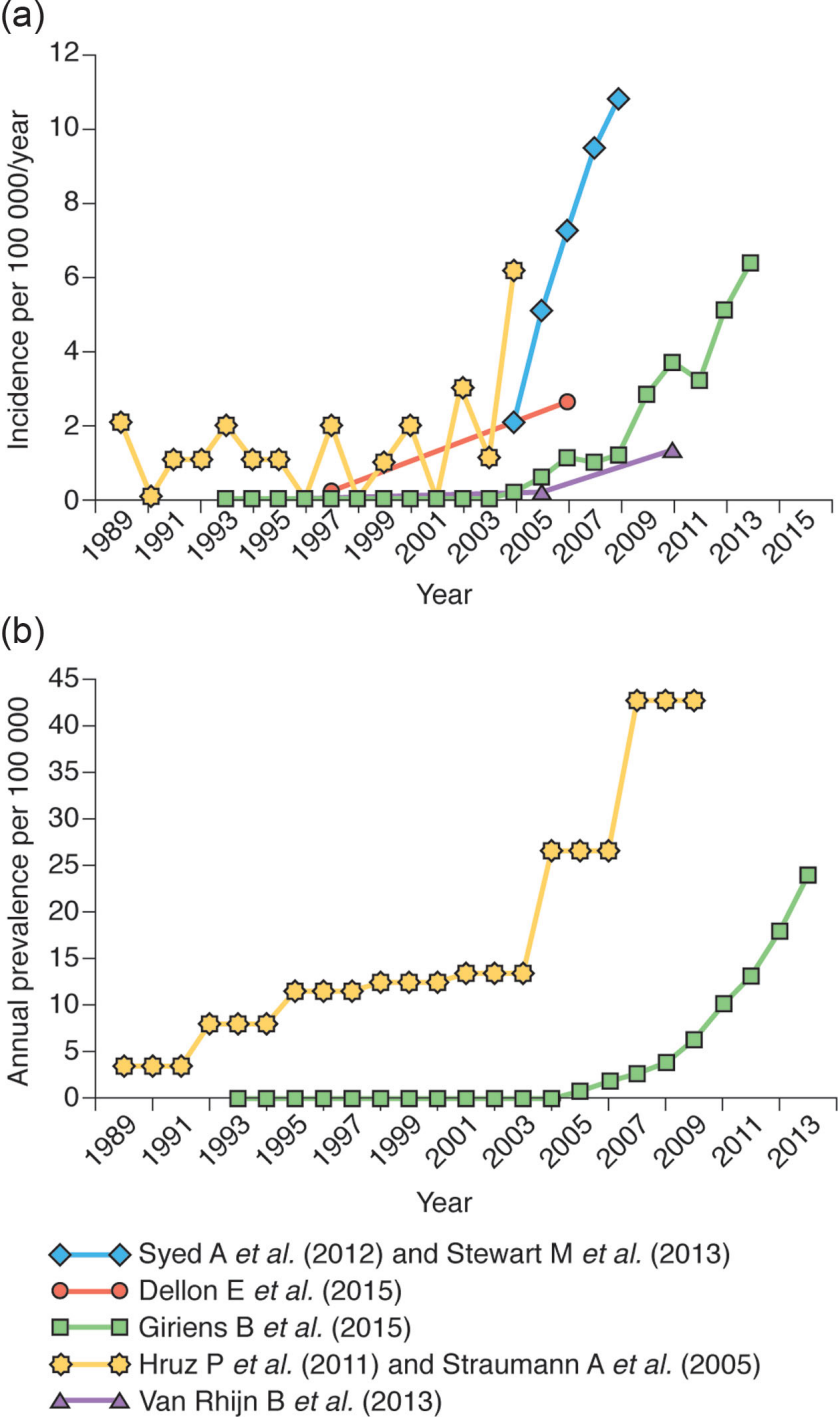
(a) Incidence and (b) prevalence of EoE over time in populations that include both children and adults. Note that populations and methods of estimation were different in the presented studies. EoE, eosinophilic esophagitis.

Seven studies reported temporal trends in the incidence and prevalence of EoE in adults and children, all of which reported an increase over time (Fig. 3).^11,12,14,17,20–22^ Three studies examined the incidence and prevalence of the disease in two regions of Switzerland.^20–22^ In one of these regions (Olten County), the prevalence increased from 2 per 100 000 in 1989 to 23 per 100 000 in 2004, and the incidence increased from 2 per 100 000 in 1989 to 7.4 per 100 000 in 2007–2009.^21,22^ In the second region (the Canton of Vaud), no inhabitants were diagnosed with EoE between 1993 and 2003.^20^ The first patients were diagnosed in 2004, and the incidence of disease increased from 0.16 per 100 000 in 2004 to 6.32 per 100 000 in 2013.^20^ The prevalence increased from 0.16 per 100 000 to 24.08 per 100 000 in the same time frame. Similarly, the incidence of the disease increased 131-fold in the Netherlands from 1996 to 2010,^17^ 20-fold in Denmark from 1997 to 2006,^14^ and 5.1-fold in Calgary, Canada from 2004 to 2008.^11,12^

### Clinical features and natural history of EoE

Evidence relating to the clinical features and natural history of EoE was available in 27 publications on studies conducted in Australia,^25^ Italy,^26,27^ Japan,^28^ Slovenia,^18^ Spain,^29^ Switzerland,^30,31^ the UK,^23^ and the USA (Table 1).^7,10,13,32–46^

**Table 1:** Summary of symptom data from natural history studies

Reference (year), country	Sample size, *n*	Ethnicity, *n* (%)	Sex, *n* (%)	Age at time of study, years, mean (SD) [median]	Diagnostic criteria^†^	Exclusion of patients with GERD or PPI-REE	Most common symptoms in total population, *n* (%)^‡^
**Children**							
Assa’ad *et al*. (2007), USA^32^	89	Ca, 84 (94) AA, 4 (5)	M, 70 (79) F, 19 (21)	NR	≥24 eos/hpf; EGD with diagnosis of EoE	NR	Emesis, 53 (60), abdominal pain, 22 (25), heartburn, 19 (21), dysphagia, 14 (16)
Cohen *et al*. (2012), UK^23^	24	NR	M, 13 (54) F, 11 (46)	NR	≥15 eos/hpf	NR	Dysphagia, 5 (21), emesis, 4 (17)
Fahey *et al.* (2017), USA^46^	36	Nonhispanic, 23 (64) Hispanic, 2 (6)	M, 29 (81) F, 7 (19)	0–21 (age at onset of symptoms: 4 (5))	>15 eos/hpf	Patients responsive to PPI excluded	Abdominal pain, 11 (31), emesis, 11 (31), poor weight gain, 6 (17), dysphagia, 6 (17)
Gill *et al*. (2007), USA^7^	44	NR	NR	9.03 (NR) [NR]	>15 eos/hpf	NR	Abdominal pain, 24 (55), emesis, 19 (43), heartburn, 17 (39), dysphagia, 4 (9)
Homan *et al*. (2015), Slovenia^18^	25	NR	M, 23 (92) F, 2 (8)	9.5 (4.71) [NR]	≥15 eos/hpf	Patients responsive to PPI excluded	Nausea and emesis, 8 (32), abdominal pain, 6 (24), food impaction, 3 (12), dysphagia, 2 (8), failure to thrive, 2 (8)
Kubik *et al.* (2017), USA^45^	251	Ca, 228 (91) AA, 16 (6)	M, 183 (73) F, 68 (27)	NR (NR) [8]	>15 eos/hpf	Patients responsive to PPI excluded	Abdominal pain, 142 (57), nausea/emesis, 122 (49), dysphagia, 109 (43), chronic cough, 50 (20)
Mansoor & Cooper (2016), USA^13^	1250	NR	NR	NR	SNOMED-CT diagnosis of ‘eosinophilic esophagitis’ with an RxNorm prescription of ‘proton-pump inhibitor’	Patients responsive to PPI excluded	GERD symptoms, 780 (62), dysphagia, 370 (30), failure to thrive, 210 (17), nausea and vomiting, 200 (16)
Moawad *et al*. (2016), USA^34^	317	Ca, 263 (83) AA, 32 (10)	M, 238 (75) F, 79 (25)	NR (NR) [8]	>15 eos/hpf	Patients responsive to PPI excluded	Data are available only for subpopulations: race (white, AA, other races) and sex (male, female)
Nethercote *et al*. (2012), Australia^25^	277^§^	NR	M, 204 (74) F, 73 (26)	7.8 (5.06) [NR]	≥15 eos/hpf	NR	Emesis, 85 (34), abdominal pain, 82 (33), diarrhoea, 57 (23), failure to thrive/weight loss, 52 (21)
Orenstein *et al*. (2000), USA^33^	21	NR	M, 17 (81) F, 4 (19)	NR	≥20 eos/hpf	NR	Food impaction, 3 (14), dysphagia, 1 (5)
Prasad *et al.* (2009), USA^10^	23	NR	M, 15 (65) F, 8 (35)	10 (6) [NR]	≥15 eos/hpf	NR	Dysphagia, 14 (61), emesis, 10 (44), abdominal pain, 7 (30), food impaction, 5 (22), GERD symptoms, 5 (22)
Rassbach *et al*. (2015), USA^37^	83	Ca, 71 (85)	M, 56 (68) F, 27 (32)	NR (NR) [10]	≥15 eos/hpf; presence of keywords ‘esophagitis’ or ‘eosinophil’ in pathology report	NR	GERD symptoms, 47 (57), emesis, 43 (52), dysphagia, 37 (45), abdominal pain, 35 (42)
Singla *et al*. (2015), USA^36^	64	Ca, 56 (88)	M, 55 (86) F, 9 (14)	8 (4.5) [NR]	≥15 eos/hpf	Patients responsive to PPI excluded	Data are available only for subpopulations: patients with inflammatory and fibrostenotic phenotypes
Spergel *et al*. (2009) USA^35^	562	Ca, 562 (90) AA, 25 (4) Asian, 15 (3)	M, 421 (75) F, 141 (25)	6.2 (NR) [NR]	20 eos/hpf; Symptomatic^¶^	Patients responsive to PPI excluded	GERD symptoms, 158 (28), failure to thrive, 118 (21), abdominal pain, 88 (16), dysphagia and food impaction, 62 (11)
Weiler *et al*. (2014), USA^38^	50	Ca, 21 (42) AA, 21 (42) Asian, 2 (4)	M, 37 (74) F, 13 (26)	NR	≥15 eos/hpf	Patients responsive to PPI excluded	GERD symptoms, 37 (74), abdominal pain, 20 (40), failure to thrive, 14 (28), dysphagia or food impaction, 11 (22)
**Adults**							
Bohm *et al.* (2017), USA^44^	58	NR	M, 37 (64) F, 21 (36)	21 (NR) [NR]	≥15 eos/hpf	NR	Abdominal pain, 31 (53), dysphagia, 19 (33), vomiting, 14 (24), heartburn/GERD, 11 (19)
Kinoshita *et al*. (2013), Japan^28^	26	NR	M, 20 (77) F, 6 (23)	49 (3) [NR]	2007 guidelines^2^ PPI-REE included	PPI-REE included (*n* = 4)	Dysphagia, 12 (46), heartburn, 2 (8)
Lipka *et al*. (2016), USA^41^	64	NR	M, 47 (73) F, 17 (27)	NR	≥15 eos/hpf	NR	Data are available only for subpopulations: patients with inflammatory and fibrostenotic phenotypes
Lynch *et al*. (2016), USA^42^	162	Ca, 150 (93)	M, 91 (56) F, 71 (44)	NR	ICD-9; met 2011 consensus guidelines^1^	NR	Data are available only for subpopulations: sex (male, female)
Mansoor & Cooper (2016), USA^13^	6590	NR	NR	NR	SNOMED-CT diagnosis of ‘eosinophilic esophagitis’ with an RxNorm prescription of ‘proton-pump inhibitor’	Patients responsive to PPI excluded	GERD symptoms, 4340 (66), dysphagia, 3810 (58), chest pain, 2000 (30), nausea and vomiting, 970 (15)
Menard-Katcher *et al*., USA^40^	53	Ca, 52 (98)	M, 40 (75) F, 13 (25)	20.5 (2.5) [NR]	>20 eos/hpf; Symptomatic^††^	NR	GERD symptoms, 33 (62), swallowing difficulties, 18 (34), dysphagia (based on MDQ-30 score), 2 (4)
Moawad *et al*. (2016), USA^34^	476	Ca, 395 (83) AA, 48 (10)	M, 328 (69) F, 148 (31)	NR (NR) [38]	>15 eos/hpf	Patients responsive to PPI excluded	Data are available only for subpopulations: race (white, AA, other races) and sex (male, female)
Prasad *et al*. (2009), USA^10^	55	NR	M, 29 (53) F, 26 (47)	37 (11) [NR]	≥15 eos/hpf	NR	Dysphagia, 51 (93), heartburn, 30 (55), food impaction, 23 (42), GERD symptoms, 21 (38)
Rassbach *et al.* (2015), USA^37^	343	Ca, 322 (94)	M, 230 (67) F, 113 (33)	NR (NR) [41]	≥15 eos/hpf; presence of keyword ‘esophagitis’ or ‘eosinophil’ in pathology report	NR	GERD symptoms, 248 (72), dysphagia, 211 (62), abdominal pain, 91 (27), emesis, 41 (12)
Savarino *et al*. (2015), Italy^26^	45	NR	M, 36 (80) F, 9 (20)	35 (16) [NR]	≥15 eos/hpf; Symptomatic^‡‡^	NR	Dysphagia (liquids and solids), 32 (71), food impaction, 29 (64), heartburn, 15 (33), GERD symptoms, 11 (24)
Savarino *et al.* (2016), Italy^27^	35	NR	M, 27 (77) F, 8 (23)	NR (age at diagnosis: 29 (NR) years)	≥15 eos/hpf; symptoms of esophageal dysfunction	Patients responsive to PPI excluded	Dysphagia, 33 (94), food impaction, 23 (66), heartburn, 9 (26), chest pain, 7 (20)
Schoepfer AM *et al*. (2013), Switzerland^30^	200	NR	M, 153 (77) F, 47 (23)	NR	≥15 eos/hpf	Patients with GERD excluded	Dysphagia, 189 (95), chest pain, 71 (36), GERD symptoms, 9 (5), abdominal pain, 3 (2)
Singla *et al*. (2015), USA^36^	191	Ca, 170 (89)	M, 134 (70) F, 57 (30)	40 (14) [NR]	≥15 eos/hpf	Patients responsive to PPI excluded	Data are available only for subpopulations: patients with inflammatory and fibrostenotic phenotypes
Straumann *et al*. (2005), Switzerland^31^	30	NR	M, 22 (73) F, 8 (27)	41 (NR) [NR]	>24 eos/hpf; history of dysphagia and food impaction; endoscopic abnormalities	NR	Dysphagia with food impaction, 30 (100), heartburn, 2 (7)
Ukleja *et al*. (2014), USA^43^	61	NR	NR	NR	≥15 eos/hpf	NR	Data are available only for subpopulations: patients who did or did not require dilations
**Adults and children**							
Castro Jimenez *et al*. (2014), Spain^29^	43	NR	M, 33 (77) F, 10 (23)	34 (13) [NR]	Esophageal dysfunction; EEACI and WAO criteria	NR	Choking, 29 (67), dysphagia, 19 (44), food impaction, 9 (21), chest pain, 4 (9), emesis, 1 (2)
DeBrosse *et al*. (2011), USA^39^	42 (rEoE)	Ca, 40 (95)	M, 29 (69) F, 13 (31)	22 (NR) [NR]	≥15 eos/hpf	NR	GERD symptoms, 34 (81), dysphagia, 21 (49), impaction, 17 (40)
Mansoor & Cooper (2016), USA^13^	7840	Ca, 7000 (89) AA, 480 (6) Asian, 440 (6)	M, 4850 (62) F, 2990 (38)	NR	SNOMED-CT diagnosis of ‘eosinophilic esophagitis’ with an RxNorm prescription of ‘proton-pump inhibitor’	Patients responsive to PPI excluded	Dysphagia, 6180 (79), GERD symptoms, 5120 (65), chest pain, 2110 (27), nausea and vomiting, 1510 (19)
Moawad *et al*. (2016), USA^34^	793	Ca, 660 (83) AA, 77 (10)	M, 571 (72) F, 222 (28)	NR (NR) [26]	>15 eos/hpf	Patients responsive to PPI excluded	Data are available only for subpopulations: race (white, AA, other races) and sex (male, female)
Rassbach *et al*. (2015), USA^37^	426	Ca, 337 (80)	M, 342 (67) F, 84 (33)	NR (NR) [38]	≥15 eos/hpf; presence of keyword ‘esophagitis’ or ‘eosinophil’ in pathology report	NR	GERD symptoms, 295 (69), dysphagia, 248 (58), abdominal pain, 126 (30), emesis, 84 (20)
Singla *et al*. (2015) USA^36^	256	Ca, 228 (89)	M, 189 (74) F, 67 (26)	32 (18) [NR]	≥15 eos/hpf	Patients responsive to PPI excluded	Data are available only for subpopulations: patients with inflammatory and fibrostenotic phenotypes

^†^Diagnostic codes were not reported unless stated in this column;

^‡^The four most common symptoms reported in a study are shown in this column. Note that in some studies fewer than four symptoms were reported. More than four symptoms are shown when several symptoms were equal in prevalence;

^§^Clinical information was available for 252 patients;^25^

^¶^At least one of the following symptoms: failure to thrive, vomiting, regurgitation, abdominal pain, food impaction, and dysphagia unresponsive to a 2-month therapeutic trial of PPIs;

^††^At least one of the following symptoms: failure to thrive, vomiting, regurgitation, abdominal pain, food impaction and dysphagia;

^‡‡^Symptoms including but not restricted to food impaction and dysphagia.

AA, African-American; Ca, Caucasian; EEACI, European Academy of Allergy and Clinical Immunology; EGD, esophagogastroduodenoscopy; EoE, eosinophilic esophagitis; eos/hpf, eosinophils per high-power field; F, female, GERD, gastroesophageal reflux disease; ICD-9, International Statistical Classification of Diseases and Related Health Problems, Ninth Revision; M, male; MDQ, Mayo Dysphagia Questionnaire; NR, not reported; PPI, proton pump inhibitor; PPI-REE, proton pump inhibitor-responsive esophageal eosinophilia; rEoE, retrospectively identified eosinophilic esophagitis; SD, standard deviation, SNOMED-CT, Systematized Nomenclature of Medicine—Clinical Terms; WAO, World Allergy Organization.

#### Patient characteristics and symptoms

**Fig. 4:**
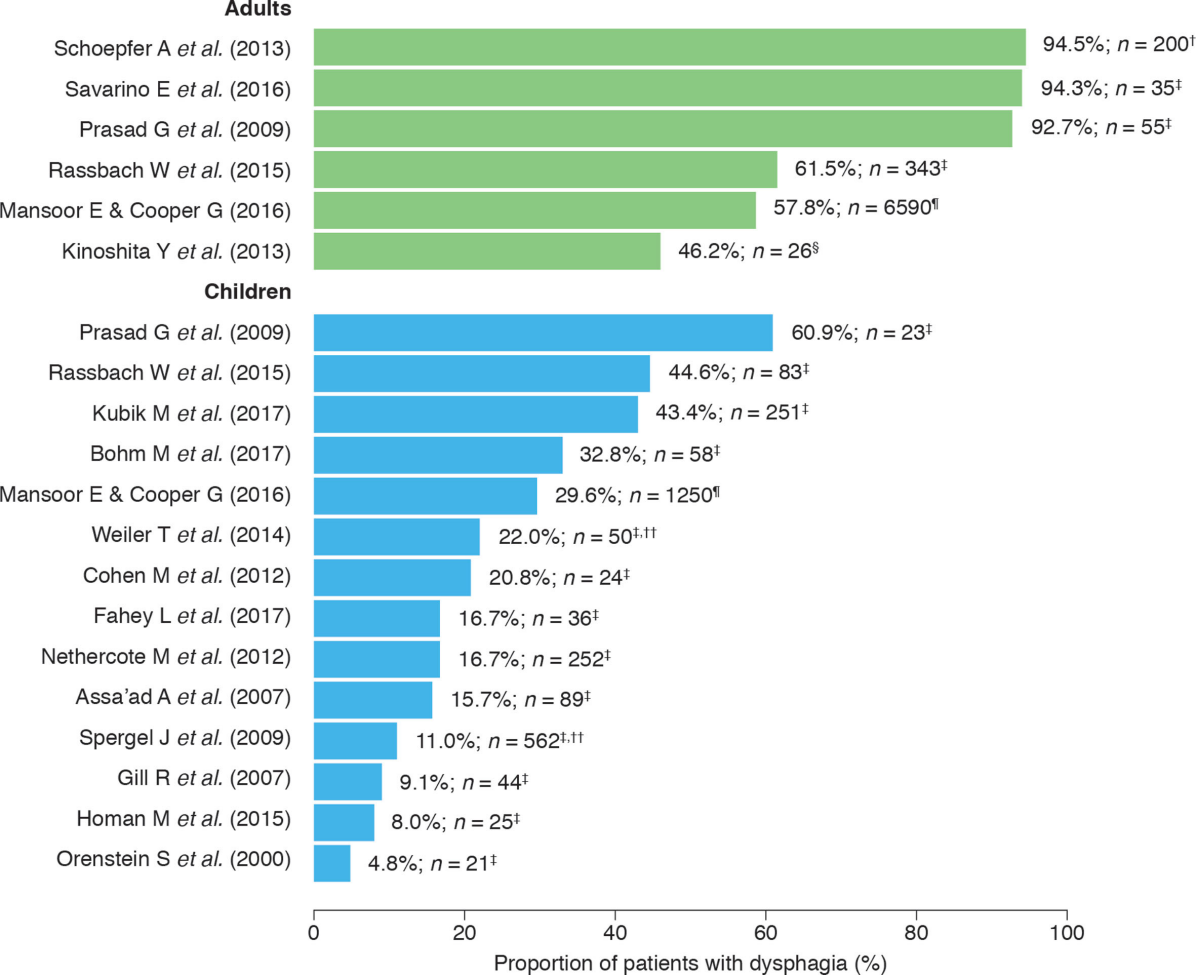
Prevalence of dysphagia in adults and children. Sample sizes (*n*) are shown to the right of each bar. Please note that the denominators of at risk populations from the studies presented in this figure may have different definitions, i.e. some populations may be highly symptomatic or selected. ^†^Diagnostic criteria based on esophageal dysfunction or esophageal eosinophilia. ^‡^Diagnostic criteria based on eosinophil count per high-power field. ^§^Diagnostic criteria based on 2007 guidelines.^2^^¶^Diagnostic criteria based on SNOMED-CT diagnosis; no diagnostic codes were reported for any other study. ^††^Data refer to dysphagia or food impaction. SNOMED-CT, Systematized Nomenclature of Medicine—Clinical Terms.

**Fig. 5:**
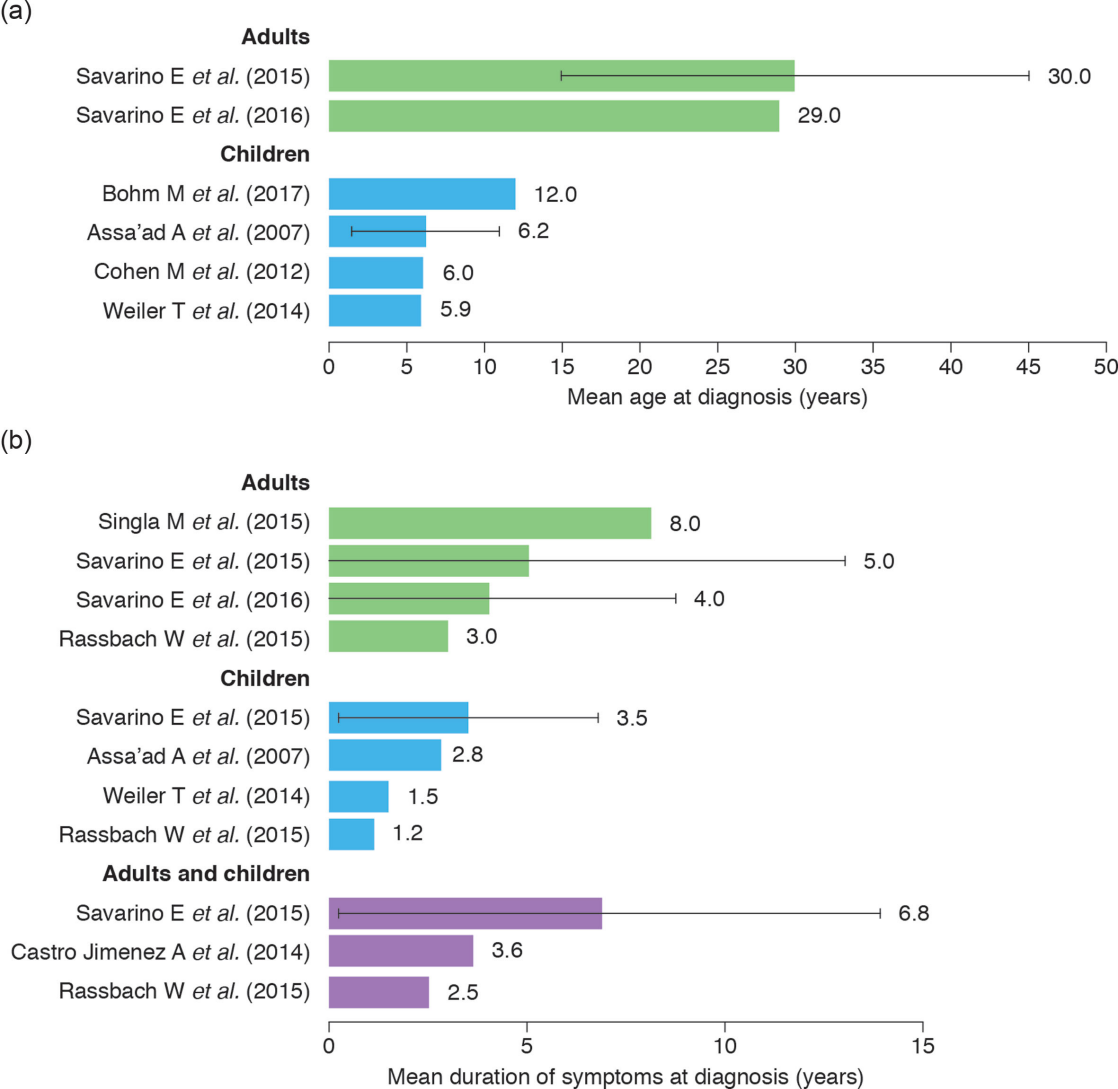
Summary of data related to (a) age at diagnosis and (b) duration of symptoms at diagnosis in children and adults. Error bars show standard deviation (the standard deviation was not reported in all of the studies). Data on mean age at diagnosis were not reported for both children and adults.

The majority of patients in the included studies were male (52.7–92.0%), and a large proportion was Caucasian (42.0–98.1%; Table 1).^7,10,13,18,23,25–46^ The most commonly reported symptoms in children were emesis (16.7–59.6%), abdominal pain (15.7–56.6%), dysphagia (4.8–60.9% of patients) (Fig. 4), and food impaction (6.7–21.7%), and in adults were dysphagia (46.2–94.5%) (Fig. 4), food impaction (16.9–65.7%), heartburn (7.7–54.5%), chest pain (0–35.5%), and acid regurgitation (4.5–38.2%).^7,10,13,18,23,25–38,41–46^ The mean age at diagnosis ranged from 5.9 to 12.0 years in children and from 29 to 30 years in adults (Fig. 5a).^23,26,27,32,38,44^ The average delay from symptom onset until diagnosis ranged from 1.2 to 3.5 years in children, from 3.0 to 8.0 years in adults, and from 2.5 to 6.8 years in populations that included both children and adults (Fig. 5b).^26,27,29,32,36–38^

#### Differences in symptom presentation

**Fig. 6:**
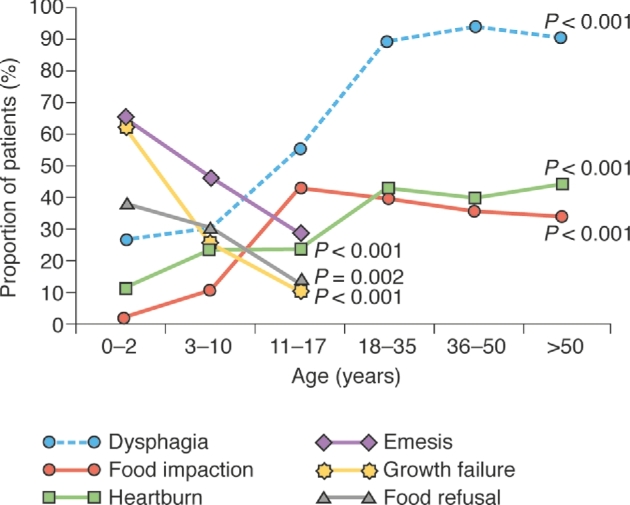
Symptoms by age category. This graph is based on data reported in Moawad *et al.*^34^

The findings from one large study of 793 children and adults demonstrated that symptom presentation differed significantly with age and race, but not with sex.^34^ This study revealed a significant positive association between age and the prevalence of dysphagia, heartburn, and food impaction, which increased from 3 years to 11–17 years for food impaction, and from 3 years to 18–35 years for heartburn and dysphagia, with all three associations plateauing thereafter (Fig. 6). Emesis, growth failure, and food refusal were significantly associated with younger age, with the peak prevalence of each occurring between 0 and 2 years of age (Fig. 6).^34^ When pediatric and adult patient groups were combined in this study, both dysphagia and food impaction were significantly more common in Caucasians than in African-Americans and other races (dysphagia—Caucasians: 74%, African-Americans: 56%, other races: 53%, *P <* 0.001; food impaction—Caucasians: 35%, African-Americans: 13%, other races: 13%, *P <* 0.001).^34^ This study also evaluated sex differences in symptoms in children and adults, and found no difference in the prevalence of dysphagia, food impaction, heartburn, or regurgitation between male and female patients.^34^ However, a second study on only adults indicated higher rates of chest pain in women than in men (27% vs. 14%, *P =* 0.03), and higher rates of dysphagia (77% vs. 62%, *P =* 0.04) and food impaction (43% vs. 28%, *P =* 0.05) in men than in women.^42^

#### Disease progression

A number of studies examined disease progression, including changes in symptoms over time, recurrence and remission of disease. These are summarized below. However, they are difficult to compare due to heterogeneity in study design, in particular whether treatment was given and, if so, whether the impact of this on disease course was assessed.

#### Changes in EoE symptoms

Three studies evaluated changes in symptoms postdiagnosis.^31,35,36^ One study of 30 adults with EoE found that after a mean follow-up of 7.2 years during which they had not been receiving medical therapy, 11 (36.7%) experienced a decrease in intensity of dysphagia, 7 (23.3%) reported a worsening in intensity of dysphagia, 11 (36.7%) described dysphagia as being stable-persistent, and one patient (3.3%) reported that symptoms of dysphagia had disappeared by the end of the follow-up period.^31^ Eleven of the patients received dilation during the study; however, it is not clear from the report how this related to the severity of their dysphagia. A second study of 562 children used retrospective and prospective medical chart review to assess changes in a subpopulation of 24 children who had not received medical or dietary treatment.^35^ After a mean follow-up period of 5.2 years, dysphagia and food impaction became worse. The third study used the Endoscopic Reference Score to classify patients into those with fibrostenotic versus inflammatory EoE.^36^ Comparison of these groups showed that a similar proportion of patients who received steroid, diet, and/or PPI therapy reported an improvement in symptoms such as dysphagia and food impaction regardless of whether they presented with fibrostenotic or inflammatory disease (69% vs. 60%, *P =* 0.256).^36^ More than half of 256 pediatric and adult patients (56%) maintained their fibrostenotic or inflammatory disease phenotype over a mean study follow-up time of 1.7 years while on therapy: 18% had remission of inflammatory EoE, 27% remained inflammatory, 29% remained fibrostenotic, 25% experienced regression of fibrostenosis, and 1% progressed from inflammatory to fibrostenotic disease.^36^

#### Recurrence of EoE

A retrospective cohort study of 78 adults and children with EoE reported a 2-year cumulative recurrence rate of dysphagia/food impaction of 31.5% (95% CI 19.6–41.7) and a 4-year cumulative recurrence rate of 49.2% (95% CI 33.6–61.1).^10^ However, it is unclear whether the patients were receiving treatment during this time frame. The authors report that 51% of patients received swallowed topical steroids for 4–8 weeks, 40% received PPIs for 6–12 weeks, and 20% were treated with medication and dilation, but did not analyze recurrence by treatment group.

#### Remission of EoE

Six studies reported on the proportion of patients achieving ‘remission’ of EoE.^18,32,33,35,36,44^ However, only three of these examined remission off treatment^18,33,35^ and only one reported the proportion of patients with both histological and symptomatic resolution off treatment.^18^ This study of 25 children with EoE in Slovenia, all of whom were treated primarily with dietary therapy, found that 20% of patients had complete remission despite food reintroduction and cessation of steroid or PPI treatment. However, it should be noted that the definitions of histological and symptomatic remission are not given in the report, and that the duration of time between treatment cessation and biopsy may have been too short to accurately assess disease remission.

#### Disease progression from childhood to adulthood

Three studies explored the progression of EoE from childhood to adulthood.^39,40,44^ In all of these studies, the current status of adults who had had EoE since childhood was assessed via a telephone survey.

In the first study,^40^ 53 adults with well-characterized EoE that had been diagnosed in childhood responded to the cross-sectional survey. At the time of the survey, their mean age was 20.5 (SD: 2.47) years and their mean age at diagnosis was 13.5 years (SD: 3.52). The survey found that two patients (4%) had a positive dysphagia score (defined as scores ≥40) on the Mayo Dysphagia Questionnaire-30 Day (MDQ-30), 18 (34%) reported some difficulty swallowing in their MDQ-30 responses, and 33 patients (62%) reported regurgitation or heartburn over the past month. During this time, 49% were receiving ongoing PPI therapy, and 76% were following an allergy-directed diet. Results from this study indicate that the majority of young adults diagnosed with EoE during childhood continue to require treatment or dietary modification for EoE during early adulthood. In a second study, adults (mean age 22 years) who had histological evidence of EoE as children were contacted a mean of 15 years after their initial childhood biopsy.^39^ At this follow-up, 49% of patients had dysphagia, 73% had recent upper gastrointestinal tract symptoms, 40% had a history of food impaction, and 41% needed ongoing care from a gastroenterologist.^39^ Again, this study suggests that symptomatology associated with EoE diagnosed in childhood commonly persists to adulthood. However, results from a third study (*N* = 58; mean duration between diagnosis and follow-up: 8.3 years) revealed that most young adults (81%) who were diagnosed with EoE as children reported resolution (47%) or improvement of their symptoms (34%).^44^ Given that within the previous 12 months, only 12% and 24% of patients were treated with steroids or PPI, respectively, the results from this study, in contrast to the previous two, suggest that a large proportion of patients had symptom resolution or improvement and did not require ongoing healthcare.

#### Association between age and endoscopic features

Four studies showed an association between older age and the presence of endoscopic features of fibrostenosis,^25,34,36,41^ while two studies found no evidence for such an association.^18,30^ In children, the prevalence of strictures significantly increased with age (<3 years vs. 3–10 years vs. >10 years, 0% vs. 1.8% vs. 6.9%, *P =* 0.04).^25^ In populations that included children and adults, the number of rings (peaked in the 18–35 years age group and plateaued thereafter, *P <* 0.001), strictures (peaked in the ≥51 years age group, *P <* 0.001), and Schatzki rings (peak in the ≥51 years age group, *P <* 0.001) increased with age, whereas white plaques decreased with age (lowest value in the ≥51 years age group, *P <* 0.001).^34^ Moreover, inflammatory endoscopic features such as plaques presented at a younger age than fibrostenotic features such as rings and strictures (24 years vs. 39 years, *P =* 0.001; 24 years vs. 34 years, *P =* 0.02).^36,41^

#### Association between diagnostic delay and endoscopic features

Data from four studies indicated an association between longer diagnostic delay and increasing presence of some endoscopic features of EoE.^30,34,36,41^ The duration of symptoms before diagnosis was significantly longer in patients with rings and strictures than in those without (rings: 24 months vs. 13 months, *P <* 0.001; strictures: 36 months vs. 15 months, *P <* 0.001).^34^ Similarly, the duration of symptoms before diagnosis was longer in patients with fibrostenotic features than in those without (8.1 years vs. 5.3 years, *P =* 0.002; 12.9 years vs. 5.1 years, *P <* 0.0001).^36,41^ Patients with a small esophageal diameter of 6.0–9.9 mm or a medium diameter of 10.0–16.9 mm had a significantly longer duration of symptoms prior to diagnosis than individuals with larger diameters of ≥17 mm (*P* < 0.0001 and *P* = 0.003, respectively).^41^

## DISCUSSION

This systematic review shows that the incidence and prevalence of EoE vary widely across North America and Europe, and have increased dramatically over time. The most common symptoms of EoE are emesis, abdominal pain, and dysphagia in children, and dysphagia, food impaction, and heartburn in adults. These symptoms differ significantly by age and race. Our review further reveals the common occurrence of delays of several years from the onset of symptoms until the diagnosis of the disease. Eosinophilic esophagitis appears to be a progressive disease that persists from childhood to adulthood. Data on the disease course are very limited. The studies that are available suggest a relatively high recurrence rate and that few patients achieve resolution without treatment.

Differences and inconsistencies in diagnostic criteria and methodology may partially explain the varying estimates of the prevalence and incidence of EoE across countries and studies. Such variance can be seen even within a single country, as was noted in the data from the Canton of Vaud compared with Olten County in Switzerland.^21,22^ One hypothesis could be that studies including patients with PPI-REE are likely to find a higher estimated prevalence and incidence than those in which such patients are excluded. However, much variation in incidence and prevalence was still seen between studies that excluded patients with PPI-REE, suggesting that factors such as differences in diagnostic criteria, diagnosis codes used to define disease, or other factors may impact the reported epidemiology of EoE.

The data also suggest that the incidence and prevalence of EoE are rising among both children and adults in developed countries. Awareness of the disease may play a crucial role in these variations, given that the lowest incidence and prevalence data are from earlier publications when less was known about the disease. However, it is unclear how much of this increase is attributable to true changes in incidence of disease. In pediatrics, variation may also be due to the increasing availability and accessibility of pediatric endoscopy equipment and expertise. Conversely, causative environmental exposures may have changed over time. The observed variations in the incidence and prevalence of EoE, and its increase over time, are consistent with observations from previous work.^47^ Moreover, it is unclear whether rates are similar in less developed countries, and whether the lack of epidemiological data in these countries is due to lack of access to technology/healthcare, due to symptoms being reported only as a nuisance and considered of low priority, or if there is a real difference in presentation and prevalence outside of the developed world.

The most commonly reported symptoms of EoE are emesis, abdominal pain, dysphagia, and food impaction in children, and dysphagia, food impaction, heartburn, and acid regurgitation in adults. It is interesting to note that dysphagia is considerably more prominent in adults than in children (46.2–94.5% vs. 4.8–60.9%), suggesting age-dependent differences in disease manifestation. However, it is important to consider the impact of language development in reported symptoms. For instance, since very young children may struggle to express complicated symptoms such as dysphagia, these symptoms may manifest themselves in food refusals for solid textures. Capturing such outcomes is difficult and may not be reflected in the data above.

The prevalence of symptoms varies significantly across studies and throughout the course of the disease, and differs significantly by age and race, while the impact of sex remains controversial.^34,42^ For instance, occurrence of dysphagia and food impaction increases considerably from early childhood, and both of these symptoms are more common in Caucasians than in African-Americans and other races.^34^ These findings suggest that heterogeneity of patient populations, particularly differences in symptom presentation depending on the age and race of the patient, may explain some of the variation in symptoms seen across studies. However, it should be noted that conclusions related to epidemiologic differences in symptomatology by race and age are limited by the lack of worldwide data.

This is the first systematic review to assess the disease course of EoE. The mean time from symptom onset to diagnosis of EoE was up to 3.5 years in children and 8.0 years in adults,^26,32,36–38^ suggesting a need for a better understanding of the common symptoms and early indicators of the disease, which would enable clinicians to provide earlier diagnosis and therefore more effective treatment as well as making patients aware of a potential underlying disease.

Early diagnosis may be particularly important because evidence from this systematic review indicates that EoE is a persistent disorder that continues from childhood to adulthood. More than a third of adult patients who were diagnosed with EoE as a child have lasting difficulties in swallowing and are in need of continuing healthcare.^39,40^ In addition, fibrostenotic features, such as rings and strictures, which are considered to be hallmarks of progression of the disease, are more prominent in older patients, suggesting that they had the disease for a number of years.^36,41^ Therefore, if fibrostenotic disease can be averted by timely treatment, delay in diagnosis may have long-lasting ramifications for patients.

This systematic review identified very limited data on the recurrence and remission rates in EoE. Furthermore, the studies that have been published in this area are very heterogeneous and difficult to interpret. More work is urgently needed in this area to guide the approach to maintenance therapy for patients with EoE.

Several strengths and limitations of this work deserve mention. Interpretation of both epidemiology and natural history data is challenged by the fact that some studies excluded patients with PPI-REE, whereas others did not specifically address this issue. Moreover, the need to account for changing disease definitions, confounders, and effect modifiers is a legitimate concern, and further analysis of epidemiologic incidence and prevalence data, particularly from population-based cohorts, is necessary. Confounders may include the availability of routine access to gastrointestinal endoscopy, as well as disease recognition, changing endoscopy practices and the work force of pediatric gastroenterologists in a given country or region. Several limitations are related to natural history data. For instance, studies concerning remission are difficult to interpret, because the definitions of remission and of the disease itself differed between studies, and very few confirmed whether resolution was histological as well as symptomatic, whether the recorded patients were undergoing treatment, and whether they were compliant with therapy. Moreover, all studies examining the effect of race and sex were undertaken in the USA, affecting the generalizability of results.

This study highlights a lack of epidemiological data for many parts of the world, including South America, Africa, and Asia. EoE is largely unstudied in these areas: only one non-English language study was identified in our searches, and inclusion of this abstract would not have impacted the conclusions presented in this systematic review. In the future, it will also be important to assess more thoroughly the incidence and prevalence estimates of EoE in specific patient subgroups to allow a better understanding of the influence of age, sex, and race on the disease. Moreover, while the prevalence and incidence of EoE appear to be increasing, the reasons behind this remain unclear. Further long-term data are needed to clarify whether the increasing incidence and prevalence of EoE result from an increasing recognition and awareness of symptoms, or instead reflect a true increase in this disease driven by some as yet unknown external factor.

Although considerable data are available on symptom presentation and disease progression of EoE, future work is needed to understand variations in symptom patterns in general and after treatment, as well as the duration and the progression of the disease. In particular, increased knowledge about whether symptoms become worse if untreated, and whether complications can be altered or prevented by maintenance therapy, is crucial for optimizing the care of patients with EoE.

In conclusion, this systematic review found that EoE is increasing in incidence and prevalence, which may be partly due to increasing recognition and awareness of symptoms. Delay in diagnosis appears to be associated with fibrostenotic disease manifestations, suggesting that timely recognition of the disease may impact its clinical course. A better understanding of the progressive nature of EoE, its symptom burden over time, and the impact of current therapies on symptom resolution are crucial in directing current clinical practice.

## Supplementary Material

Supplemental data

## Notes

Specific author contributions: Study concept and design: Nicholas J. Shaheen, Vincent Mukkada, Christian S. Eichinger, Helen Schofield, Lora Todorova, Gary W. Falk; Analysis and interpretation of data: Nicholas J. Shaheen, Vincent Mukkada, Christian S. Eichinger, Helen Schofield, Lora Todorova, Gary W. Falk; Critical revision of the manuscript for important intellectual content: Nicholas J. Shaheen, Vincent Mukkada, Lora Todorova, Gary W. Falk; Acquisition of data by carrying out the systematic literature searches: Christian S. Eichinger, Helen Schofield; Drafting of the manuscript: Christian S. Eichinger. All authors are responsible for the content and the decision to submit the manuscript. All authors approved the final version of this manuscript, including the authorship list.

## References

[1] Liacouras C A, Furuta G T, Hirano I Eosinophilic esophagitis: updated consensus recommendations for children and adults. Aliment Pharmacol Ther 2011; 128: 3–15. 10.1016/j.jaci.2011.02.040 21477849

[2] Furuta G T, Liacouras C A, Collins M H Eosinophilic esophagitis in children and adults: a systematic review and consensus recommendations for diagnosis and treatment. Gastroenterology 2007; 133: 1342–63. 17919504 10.1053/j.gastro.2007.08.017

[3] Lucendo A J, Molina-Infante J, Arias A Guidelines on eosinophilic esophagitis: evidence-based statements and recommendations for diagnosis and management in children and adults. United European Gastroenterol J 2017; 5: 335–58. 10.1177/2050640616689525 PMC5415218 28507746

[4] Liberati A, Altman D G, Tetzlaff J The PRISMA statement for reporting systematic reviews and meta-analyses of studies that evaluate healthcare interventions: explanation and elaboration. BMJ 2009; 339: b2700. 19622552 10.1136/bmj.b2700 PMC2714672

[5] Ally M R, Maydonovitch C L, Betteridge J D, Veerappan G R, Moawad F J Prevalence of eosinophilic esophagitis in a United States military health-care population. Dis Esophagus 2015; 28: 505–11. 24827543 10.1111/dote.12229

[6] Dellon E S, Jensen E T, Martin C F, Shaheen N J, Kappelman M D Prevalence of eosinophilic esophagitis in the United States. Clin Gastroenterol Hepatol 2014; 12: 589–96. 24035773 10.1016/j.cgh.2013.09.008 PMC3952040

[7] Gill R, Durst P, Rewalt M, Elitsur Y Eosinophilic esophagitis disease in children from West Virginia: a review of the last decade (1995–2004). Am J Gastroenterol 2007; 102: 2281–85. 17573789 10.1111/j.1572-0241.2007.01352.x

[8] Kim S, Kim S, Sheikh J Prevalence of eosinophilic esophagitis in a population-based cohort from Southern California. J Allergy Clin Immunol Pract 2015; 3: 978–79. 26164810 10.1016/j.jaip.2015.06.008

[9] Noel R J, Putnam P E, Rothenberg M E Eosinophilic esophagitis. N Engl J Med 2004; 351: 940–41. 15329438 10.1056/NEJM200408263510924

[10] Prasad G A, Alexander J A, Schleck C D Epidemiology of eosinophilic esophagitis over three decades in Olmsted County, Minnesota Clin Gastroenterol Hepatol 2009; 7: 1055–61. 19577011 10.1016/j.cgh.2009.06.023 PMC3026355

[11] Stewart M J, Shaffer E, Urbanski S J, Beck P L, Storr M A The association between celiac disease and eosinophilic esophagitis in children and adults. BMC Gastroenterol 2013; 13: 96. 23721294 10.1186/1471-230X-13-96 PMC3682941

[12] Syed A A, Andrews C N, Shaffer E, Urbanski S J, Beck P, Storr M The rising incidence of eosinophilic oesophagitis is associated with increasing biopsy rates: a population-based study. Aliment Pharmacol Ther 2012; 36: 950–58. 22994460 10.1111/apt.12053

[13] Mansoor E, Cooper G S The 2010–2015 Prevalence of eosinophilic esophagitis in the USA: a population-based study. Dig Dis Sci 2016; 61: 2928–34. 27250980 10.1007/s10620-016-4204-4 PMC5021560

[14] Dellon E S, Erichsen R, Baron J A The increasing incidence and prevalence of eosinophilic oesophagitis outpaces changes in endoscopic and biopsy practice: national population-based estimates from Denmark. Aliment Pharmacol Ther 2015; 41: 662–70. 25684441 10.1111/apt.13129 PMC4504237

[15] Dalby K, Nielsen R G, Kruse-Andersen S Eosinophilic oesophagitis in infants and children in the region of southern Denmark: a prospective study of prevalence and clinical presentation. J Pediatr Gastroenterol Nutr 2010; 51: 280–2. 20512060 10.1097/MPG.0b013e3181d1b107

[16] O’Donnell S, Kelly O B, Breslin N Eosinophilic oesophagitis: an Irish experience. Eur J Gastroenterol Hepatol 2011; 23: 1116–21. 21946074 10.1097/MEG.0b013e32834a5870

[17] van Rhijn B D, Verheij J, Smout A J, Bredenoord A J Rapidly increasing incidence of eosinophilic esophagitis in a large cohort. Neurogastroenterol Motil 2013; 25: 47–52. 22963642 10.1111/nmo.12009

[18] Homan M, Blagus R, Jeverica A K, Orel R Pediatric eosinophilic esophagitis in Slovenia: data from a retrospective 2005–2012 epidemiological study. J Pediatr Gastroenterol Nutr 2015; 61: 313–18. 26020481 10.1097/MPG.0000000000000797

[19] Arias A, Lucendo A J Prevalence of eosinophilic oesophagitis in adult patients in a central region of Spain. Eur J Gastroenterol Hepatol 2013; 25: 208–12. 23075697 10.1097/MEG.0b013e32835a4c95

[20] Giriens B, Yan P, Safroneeva E Escalating incidence of eosinophilic esophagitis in Canton of Vaud, Switzerland, 1993–2013: a population-based study. Allergy 2015; 70: 1633–39. 26304142 10.1111/all.12733

[21] Hruz P, Straumann A, Bussmann C Escalating incidence of eosinophilic esophagitis: a 20-year prospective, population-based study in Olten County, Switzerland. J Allergy Clin Immunol 2011; 128: 1349–50. 22019091 10.1016/j.jaci.2011.09.013

[22] Straumann A, Simon H U Eosinophilic esophagitis: escalating epidemiology? J Allergy Clin Immunol 2005; 115: 418–19. 15696105 10.1016/j.jaci.2004.11.006

[23] Cohen M C, Rao P, Thomson M, Al-Adnani M Eosinophils in the oesophageal mucosa: clinical, pathological and epidemiological relevance in children: a cohort study. BMJ Open 2012; 2: e000493. 10.1136/bmjopen-2011-000493 PMC3278487 22240650

[24] Cherian S, Smith N M, Forbes D A Rapidly increasing prevalence of eosinophilic oesophagitis in Western Australia. Arch Dis Child 2006; 91: 1000–4. 16877474 10.1136/adc.2006.100974 PMC2083004

[25] Nethercote M, Heine R G, Kansal S Clinical features of eosinophilic esophagitis in a consecutive series of pediatric patients in an Australian tertiary referral center. J Food Allergy 2012; 1: 160–69.

[26] Savarino E, Tolone S, Caccaro R Clinical, endoscopic, histological and radiological characteristics of Italian patients with eosinophilic oesophagitis. Dig Liver Dis 2015; 47: 1033–38. 26377770 10.1016/j.dld.2015.08.013

[27] Savarino E V, Tolone S, Bartolo O The GerdQ questionnaire and high resolution manometry support the hypothesis that proton pump inhibitor-responsive oesophageal eosinophilia is a GERD-related phenomenon. Aliment Pharmacol Ther 2016; 44: 522–30. 27373195 10.1111/apt.13718

[28] Kinoshita Y, Furuta K, Ishimaura N Clinical characteristics of Japanese patients with eosinophilic esophagitis and eosinophilic gastroenteritis. J Gastroenterol 2013; 48: 333–39. 22847555 10.1007/s00535-012-0640-x

[29] Castro Jimenez A, Gomez Torrijos E, Garcia Rodriguez R Demographic, clinical and allergological characteristics of eosinophilic esophagitis in a Spanish central region. Allergol Immunopathol (Madr) 2014; 42: 407–14. 23845923 10.1016/j.aller.2013.04.004

[30] Schoepfer A M, Safroneeva E, Bussmann C Delay in diagnosis of eosinophilic esophagitis increases risk for stricture formation in a time-dependent manner. Gastroenterology 2013; 145: 1230–36. 23954315 10.1053/j.gastro.2013.08.015

[31] Straumann A, Spichtin H P, Grize L, Bucher K A, Beglinger C, Simon H U Natural history of primary eosinophilic esophagitis: a follow-up of 30 adult patients for up to 11.5 years. Gastroenterology 2003; 125: 1660–9. 14724818 10.1053/j.gastro.2003.09.024

[32] Assa’ad A H, Putnam P E, Collins M H Pediatric patients with eosinophilic esophagitis: an 8-year follow-up. J Allergy Clin Immunol 2007; 119: 731–38. 17258309 10.1016/j.jaci.2006.10.044

[33] Orenstein S R, Shalaby T M, Di Lorenzo C The spectrum of pediatric eosinophilic esophagitis beyond infancy: a clinical series of 30 children. Am J Gastroenterol 2000; 95: 1422–30. 10894574 10.1111/j.1572-0241.2000.02073.x

[34] Moawad F J, Dellon E S, Achem S R Effects of race and sex on features of eosinophilic esophagitis. Clin Gastroenterol Hepatol 2016; 14: 23–30. 26343181 10.1016/j.cgh.2015.08.034

[35] Spergel J M, Brown-Whitehorn T F, Beausoleil J L 14 years of eosinophilic esophagitis: clinical features and prognosis. J Pediatr Gastroenterol Nutr 2009; 48: 30–36. 19172120 10.1097/MPG.0b013e3181788282

[36] Singla M B, Chehade M, Brizuela D Early comparison of inflammatory vs. fibrostenotic phenotype in eosinophilic esophagitis in a multicenter longitudinal study. Clin Transl Gastroenterol 2015; 6: e132. 26680264 10.1038/ctg.2015.62 PMC4816096

[37] Rassbach W, Rubenstein J H, Elkins M, DeMatos V, Greenson J K, Greenhawt M Age-based differences in the diagnosis and management of esophageal eosinophilia. J Allergy Clin Immunol Pract 2015; 3: 81–7. 25577623 10.1016/j.jaip.2014.06.019 PMC7423520

[38] Weiler T, Mikhail I, Singal A, Sharma H Racial differences in the clinical presentation of pediatric eosinophilic esophagitis. J Allergy Clin Immunol Pract 2014; 2: 320–25. 24811024 10.1016/j.jaip.2014.01.011

[39] DeBrosse C W, Franciosi J P, King E C Long-term outcomes in pediatric-onset esophageal eosinophilia. J Allergy Clin Immunol 2011; 128: 132–38. 21636117 10.1016/j.jaci.2011.05.006 PMC3130990

[40] Menard-Katcher P, Marks K L, Liacouras C A, Spergel J M, Yang Y X, Falk G W The natural history of eosinophilic oesophagitis in the transition from childhood to adulthood. Aliment Pharmacol Ther 2013; 37: 114–21. 23121227 10.1111/apt.12119

[41] Lipka S, Kumar A, Richter J E Impact of diagnostic delay and other risk factors on eosinophilic esophagitis phenotype and esophageal diameter. J Clin Gastroenterol 2016; 50: 134–40. 25710524 10.1097/MCG.0000000000000297

[42] Lynch K L, Dhalla S, Chedid V Gender is a determinative factor in the initial clinical presentation of eosinophilic esophagitis. Dis Esophagus 2016; 29: 174–8. 25626069 10.1111/dote.12307

[43] Ukleja A, Shiroky J, Agarwal A, Allende D Esophageal dilations in eosinophilic esophagitis: a single center experience. World J Gastroenterol 2014; 20: 9549–55. 25071351 10.3748/wjg.v20.i28.9549 PMC4110588

[44] Bohm M, Jacobs J W Jr, Gupta A, Gupta S, Wo J M Most children with eosinophilic esophagitis have a favorable outcome as young adults. Dis Esophagus 2017; 30: 1–6. 10.1111/dote.12454 26822685

[45] Kubik M, Thottam P, Shaffer A, Choi S The role of the otolaryngologist in the evaluation and diagnosis of eosinophilic esophagitis. Laryngoscope 2017; 127: 1459–64. 27900765 10.1002/lary.26373

[46] Fahey L, Robinson G, Weinberger K, Giambrone A E, Solomon A B Correlation between aeroallergen levels and new diagnosis of eosinophilic esophagitis in New York City. J Pediatr Gastroenterol Nutr 2017; 64: 22–25. 27111345 10.1097/MPG.0000000000001245 PMC5074923

[47] Arias A, Perez-Martinez I, Tenias J M, Lucendo A J Systematic review with meta-analysis: the incidence and prevalence of eosinophilic oesophagitis in children and adults in population-based studies. Aliment Pharmacol Ther 2016; 43: 3–15. 10.1111/apt.13441 26510832

