# Supplementary material for: Natural history of eosinophilic esophagitis: a systematic review of epidemiology and disease course
Source: Dis Esophagus. 2018 Mar 31;31(8):doy015. doi: 10.1093/dote/doy015 (PMC6102800; doi:10.1093/dote/doy015)
Supplement: Supplemental data [file doy015_supplementary_files.docx]

## Supplementary Table 1 Search strings

***Ovid MEDLINE In-process & Other Non-indexed Citations, Ovid MEDLINE Daily, and Ovid MEDLINE 1946 to present***

| **#** | **Searches** |
| --- | --- |
| 1 | eosinophilic esophagitis/ or eosinophilic esophagitis.mp. or eosinophilic oesophagitis.mp. or allergic oesophagitis.mp. |
| 2 | disease progression.mp. or clinical progression.tw. or clinical course.tw. or clinical history.tw. or natural progression.tw. or natural course.tw. or natural history.tw. or disease progression.tw. or disease course.tw. or disease history.tw. or incidence.tw. or prevalence.tw. |
| 3 | exp natural history/ |
| 4 | exp epidemiology/ |
| 5 | (incidence adj5 (eosinophilic esophagitis or eosinophilic oesophagitis or allergic esophagitis or allergic oesophagitis or disease)).mp. |
| 6 | (prevalence adj5 (eosinophilic esophagitis or eosinophilic oesophagitis or allergic esophagitis or allergic oesophagitis or disease)).mp. |
| 7 | (life expectancy or survival).mp. |
| 8 | or/2-7 |
| 9 | 1 and 8 |
| 10 | (animals not (humans and animals)).mp. |
| 11 | 9 not 10 |

***Embase 1974 to present***

| **#** | **Searches** |
| --- | --- |
| 1 | eosinophilic esophagitis/ or eosinophilic oesophagitis/ or allergic esophagitis/ or eosinophilic esophagitis.mp. or eosinophilic oesophagitis.mp. or allergic oesophagitis.mp. |
| 2 | disease progression.mp. or clinical progression.tw. or clinical course.tw. or clinical history.tw. or natural progression.tw. or natural course.tw. or natural history.tw. or disease progression.tw. or disease course.tw. or disease history.tw. or incidence.tw. or prevalence.tw. |
| 3 | exp natural history/ |
| 4 | exp epidemiology/ |
| 5 | (incidence adj5 (eosinophilic esophagitis or eosinophilic oesophagitis or allergic esophagitis or allergic oesophagitis or disease)).mp. |
| 6 | (prevalence adj5 (eosinophilic esophagitis or eosinophilic oesophagitis or allergic esophagitis or allergic oesophagitis or disease)).mp. |
| 7 | (life expectancy or survival).mp. |
| 8 | or/2-7 |
| 9 | 1 and 8 |
| 10 | (animals not (humans and animals)).mp. |
| 11 | 9 not 10 |

***All Evidence-Based Medicine Reviews − CDSR, ACP Journal Club Archives, DARE, CCTR, CMR, HTA Database, and NHSEED***

| **#** | **Searches** |
| --- | --- |
| 1 | eosinophilic esophagitis/ or eosinophilic esophagitis.mp. or eosinophilic oesophagitis.mp. or allergic oesophagitis.mp. |
| 2 | disease progression.mp. or clinical progression.tw. or clinical course.tw. or clinical history.tw. or natural progression.tw. or natural course.tw. or natural history.tw. or disease progression.tw. or disease course.tw. or disease history.tw. or incidence.tw. or prevalence.tw. |
| 3 | exp natural history/ |
| 4 | exp epidemiology/ |
| 5 | (incidence adj5 (eosinophilic esophagitis or eosinophilic oesophagitis or allergic esophagitis or allergic oesophagitis or disease)).mp. |
| 6 | (prevalence adj5 (eosinophilic esophagitis or eosinophilic oesophagitis or allergic esophagitis or allergic oesophagitis or disease)).mp. |
| 7 | (life expectancy or survival).mp. |
| 8 | or/2-7 |
| 9 | 1 and 8 |
| 10 | (animals not (humans and animals)).mp. |
| 11 | 9 not 10 |

ACP, American College of Physicians; CCTR, Cochrane Central Register of Controlled Trials; CDSR, Cochrane Database of Systemic Reviews; CMR, Cochrane Methodology Register; DARE, Database of Abstracts of Reviews of Effects; HTA, Health Technology Assessment; NHSEED, National Health Service Economic Evaluation Database.

## Supplementary Table 2 Eligibility criteria in PICOS format

|  | **Inclusion criteria** | **Exclusion criteria** |
| --- | --- | --- |
| Population | Adults or children with a diagnosis of EoE,  (defined as an eosinophil count ≥ 15 eos/hpf, or a positive diagnostic code [e.g. ICD-9, ICD-10])^†^ |  |
| Intervention and comparators | Epidemiology   - Not restricted by intervention | Natural history   - Intervention studies |
| Outcomes | Epidemiology   - Incidence - Prevalence - Risk factors - Trend analysis   Natural history   - Major and minor disease features - Function abilities (e.g. physical, cognitive, and psychiatric) - Severity of manifestations - Time course of manifestation progression - Frequency of manifestations - Predictors of progression - Survival | Natural history   - Studies with < 20 patients |
| Study design | Epidemiology   - Cohort - Case–control - Cross-sectional - Retrospective chart reviews   Natural history^‡^   - Published medical literature review - Retrospective chart review - Prospective cross-sectional - Prospective longitudinal | Epidemiology   - Non-population-based studies |
| Publication date | Not restricted |  |
| Language | English language only (foreign publications with an English abstract considered for inclusion) |  |

^†^Studies were included regardless of whether they stated that they had excluded individuals with symptoms of GERD or patients who were responsive to proton pump inhibitor (PPI) treatment. ^‡^Studies reporting data from the same data source (e.g. registry or hospital) were reviewed and were only included if the two studies reported different information. If there was complete overlap, the study reporting on the largest population of patients or from the most complete data set was included and the other study was excluded. If there was only partial overlap, both papers were included; however, the overlapping data were extracted from the paper with the largest patient population.

EoE, eosinophilic esophagitis; eos/hpf, eosinophils per high-power field.

## Supplementary Table 3 Summary of outcome data from epidemiology studies

| **Reference (year)** | **Population** | **Time period of estimate** | **Sample**  **size** | **Diagnostic criteria** | **Exclusion**  **criteria** | **Incidence of EoE per 100 000^†^** | **Prevalence of EoE per 100 000^†^** |
| --- | --- | --- | --- | --- | --- | --- | --- |
| **USA and Canada** | | | | | | | |
| Ally M *et al.* (2015)^5^ | Adults and children | 2008–2009 | 10 180 515 | ICD-9 code  (≥ 2 claims in the same calendar year) | No exclusions reported | NR | 9.7 |
| Dellon E *et al.* (2014)^6^ | Adults and children | 2009–2011 | 11 569 217 | ICD-9 code  (single instance) | No exclusions reported | NR | 56.7 |
| Gill R *et al.* (2007)^7^ | Children | 1995–2004 | ~600 000 | > 15 eos/hpf | Histological reports consistent with diagnoses of eosinophilic gastroenteritis, Crohn’s or celiac disease | NR | 7.3 |
| Kim S *et al.* (2015)^8^ | Adults and children | 2008–2013 | 3 486 069 | ICD-9 code  (number of instances not specified) | No exclusions reported | NR | 45 |
| Mansoor E & Cooper G (2016)^13^ | Adults and children | 2010–2015 | 30 301 440 | SNOMED-CT diagnosis of ‘‘eosinophilic esophagitis’’ with an RxNorm prescription of ‘‘proton-pump inhibitor’ | Patients responsive to PPI | NR | 25.9 |
| Noel R *et al.* (2004)^9^ | Children | 2000–2003 | – | ≥ 24 eos/hpf | Eosinophilia in any other intestinal segment | Range, 9.1–12.8 | Range,  9.9–43.0 |
| Prasad G *et al.* (2009)^10^ | Adults and children | 1976–2005 | ~120 000 | ≥ 15 eos/hpf | No exclusions reported | 2.4^‡^ | 54.0^‡^ |
| Stewart M *et al.* (2013)^11^ and Syed A *et al.* (2012)^12^ | Adults and children | 2004–2008 | 1 200 000 | > 15 eos/hpf | No exclusions reported;^11^ esophageal biopsies on surgically obtained specimens were excluded^12^ | Range, 2.1–10.7 | NR |
| **Europe** | | | | | | | |
| Arias A *et al.* (2013)^19^ | Adults | 2005–2011 | 89 642 | ≥ 15 eos/hpf | Patients responsive to PPI; GERD, eosinophilia in gastric and duodenal mucosa, and other causes of esophageal eosinophilia | 6.4 | 44.6 |
| Cohen M *et al.* (2012)^23^ | Children | 2007–2008 | NR | ≥ 15 eos/hpf | Involvement of other parts of the gastrointestinal tract | 4.5 | NR |
| Dalby K *et al.* (2010)^15^ | Children | 2005–2007 | 256 164 | ≥ 15 eos/hpf | Patients responsive to PPI, and those with diseases known to cause secondary symptoms of GERD | 1.6 | NR |
| Dellon E *et al.* (2015)^14^ | Adults and children | 1997–2012 | NR | Approx. ≥ 15 eos/hpf;  SNOMED and ICD-10 code (number of instances not specified) | Patients were excluded if they had 1 of 11 ICD-10 codes for disorders that could explain esophageal eosinophilia from a secondary cause | Range, 0.1–2.6 | 13.8 |
| Giriens B *et al.* (2015)^20^ | Adults and children | 1993–2013 | 743 317 | ≥ 15 eos/hpf | Patients responsive to PPI and those without PPI trial, and patients with GERD and disorders that could cause esophageal eosinophilia | Range,  0.0–6.3 | Range,  0.0–24.1 |
| Homan M *et al.* (2015)^18^ | Children | 2005–2012 | NR | ≥ 15 eos/hpf | Patients responsive to PPI, and patients with disorders that could cause esophageal eosinophilia | 0.8  Range, 0.2–18 | NR |
| Hruz P *et al.* (2011)^21^ and Straumann *et al.* (2005)^22^ | Adults and children | 1989–2009 | ~90 000/  100 000 | ≥ 24 eos/hpf | Patients responsive to PPI, and patients with disorders that could cause esophageal eosinophilia;^21^ patients responsive to PPI^22^ | 2.5  Range,  1.2–7.4 | Range,  3.6–42.8 |
| O'Donnell S *et al.* (2011)^16^ | Adults and children | 2000–2008 | 350 000 | > 15 eos/hpf | EoE was confirmed based on expert opinion of symptoms suggestive of EoE (may include GERD) | 5/1000 person-years^§^ | NR |
| Van Rhijn B *et al.* (2013)^17^ | Adults and children | 1996–2010 | 16 615 394 | ≥ 15 eos/hpf | Unclear | 0.3  Range,  0.01–1.3 | NR |
| **Australia** | | | | | | | |
| Cherian S *et al.* (2006)^24^ | Children | 1995, 1999, 2004 | NR | > 24 eos/hpf | Structural abnormalities of esophagus, celiac disease, inflammatory bowel disease, or multiple biopsies in same year | NR | Range,  0.5–8.9 |

^†^The ranges shown indicate the lowest and highest annual prevalence or incidence values reported in the given time period of estimate.

^‡^Age- and sex-adjusted values are reported.

^§^This incidence rate refers to esophageal biopsies (*n* = 11 072) rather than the approximate catchment population (*n* = 350 000) of the hospital.

EoE, eosinophilic esophagitis; eos/hpf, eosinophils per high-power field; GERD, gastroesophageal reflux disease; ICD-9/10, International Statistical Classification of Diseases and Related Health Problems, Ninth/Tenth Revision; NR, not reported, PPI, proton pump inhibitor; SNOMED-CT, Systematized Nomenclature of Medicine – Clinical Terms.

## Supplementary Table 4 Quality assessment of all identified studies

| **Reference (year)** | **Study design** | **Quality of evidence** | | | | | | | | | | | | | | | | |
| --- | --- | --- | --- | --- | --- | --- | --- | --- | --- | --- | --- | --- | --- | --- | --- | --- | --- | --- |
|  | **Cohort studies** | **Selection** | | | | | | **Comparability** | | **Outcome** | | | | | | | **Total score** | |
|  |  | **Representativeness of the exposed cohort:**  a) truly* representative  b) somewhat representative*  c) selected group of patients  d) no description of the derivation of the cohort | **Selection of the non-exposed cohort:**  a) drawn from same community as intervention cohort*  b) drawn from a different source  c) no description of the derivation of the non-intervention cohort | | **Ascertainment of exposure:**  a) secure record*  b) structured interview*  c) written self-report  d) other / no description | **Demonstration that outcome of interest was not present at start of study:**  a) yes*  b) no | | **Comparability of cohorts on the basis of the design or analysis controlled for confounders:**  a) study controls for age and sex*  b) study controls for any additional factors* | | **Assessment of outcome:**  a) independent blind assessment*  b) record linkage*  c) self-report  d) other / no description | | **Was follow-up long enough for outcomes to occur:**  a) yes, if median duration of follow-up >= 6 month*  b) no, if median duration of follow-up < 6 months | | | **Adequacy of follow-up of cohorts:**  a) complete follow up: all subjects accounted for*  b) subjects lost to follow up unlikely to introduce bias: number lost <= 20%, or description of those lost suggesting no different from those followed*  c) follow-up rate < 80% and no description of those lost  d) no statement or unclear | |  | |
| **Natural history studies** | | | | | | | | | | | | | | | | | | |
| Assa’ad A (2007)^32^ | Retrospective database analysis | a (+1) | NA (0) | | a (+1) | NA (0) | | NA (0) | | b (+1) | | a (+1) | | | b (+1) | | 5/8  Good | |
| Bohm M (2017)^44^ | Prospective registry study and phone survey | a (+1) | NA (0) | | a (+1) | NA (0) | | NA (0) | | b and c (+1) | | a (+1) | | | d (0) | | 4/8  Satisfactory | |
| Cohen M (2012)^23^ | Retrospective chart review | a (+1) | NA (0) | | a (+1) | NA (0) | | NA (0) | | b (+1) | | a (+1) | | | d (0) | | 4/8  Satisfactory | |
| DeBrosse C (2011)^39^ | Case control study | a (+1) | NA (0) | | a (+1) | NA (0) | | a (+1) | | b (+1) | | a (+1) | | | b (+1) | | 6/8  Good | |
| Fahey L (2017)^46^ | Retrospective chart review | a (+1) | NA (0) | | a (+1) | NA (0) | | NA (0) | | b (+1) | | NA (0) | | | NA (0) | | 5/8  Good | |
| Gill R (2007)^7^ | Retrospective review | a (+1) | NA (0) | | a (+1) | NA (0) | | NA (0) | | b (+1) | | a (+1) | | | c (0) | | 4/8  Satisfactory | |
| Homan M (2015)^18^ | Retrospective cohort study | a (+1) | NA (0) | | a (+1) | NA (0) | | NA (0) | | b (+1) | | b (0) | | | c (0) | | 3/8  Poor | |
| Kubik M (2017)^45^ | Retrospective chart review | a (+1) | NA (0) | | a (+1) | NA (0) | | NA (0) | | b (+1) | | a (+1) | | | d (0) | | 4/8  Satisfactory | |
| Lipka S (2016)^41^ | Retrospective cohort study | a (+1) | NA (0) | | a (+1) | NA (0) | | NA (0) | | b (+1) | | a (+1) | | | d (0) | | 4/8  Satisfactory | |
| Lynch K (2016)^42^ | Retrospective review | a (+1) | NA (0) | | a (+1) | NA (0) | | NA (0) | | b (+1) | | NA (0) | | | d (0) | | 3/8  Poor | |
| Mansoor E & Cooper G (2016)^13^ | Retrospective database review | a (+1) | NA (0) | | a (+1) | NA (0) | | a (+1) | | b (+1) | | NA (0) | | | d (0) | | 4/8  Satisfactory | |
| Nethercote M (2012)^25^ | Retrospective chart review | a (+1) | NA (0) | | a (+1) | NA (0) | | NA (0) | | b (+1) | | a (+1) | | | d (0) | | 4/8  Satisfactory | |
| Orenstein S (2000)^33^ | Clinical series, retrospective review and prospective interview | a (+1) | NA (0) | | a and b (+1) | NA (0) | | NA (0) | | b and c (+1) | | NA (0) | | | d (0) | | 3/8  Poor | |
| Prasad G (2009)^10^ | Retrospective review and prospective interview | a (+1) | NA (0) | | a and b (+1) | NA (0) | | NA (0) | | b (+1) | | a (+1) | | | d (0) | | 4/8  Satisfactory | |
| Rassbach W (2015)^37^ | Retrospective cohort study | a (+1) | NA (0) | | a (+1) | NA (0) | | NA (0) | | b (+1) | | a (+1) | | | d (0) | | 4/8  Satisfactory | |
| Savarino E (2015)^26^ | Prospective cohort study | a (+1) | NA (0) | | b (+1) | NA (0) | | NA (0) | | b (+1) | | NA (0) | | | d (0) | | 4/8  Poor | |
| Savarino E (2016)^27^ | Prospective cohort study | a (+1) | NA (0) | | a (+1) | NA (0) | | NA (0) | | b (+1) | | NA (0) | | | d (0) | | 4/8  Satisfactory | |
| Schoepfer AM (2013)^30^ | Retrospective database review | a (+1) | NA (0) | | a (+1) | NA (0) | | NA (0) | | b (+1) | | a (+1) | | | d (0) | | 4/8  Satisfactory | |
| Singla M *(*2015)^36^ | Prospective case series | a (+1) | NA (0) | | a (+1) | NA (0) | | NA (0) | | b (+1) | | a (+1) | | | d (0) | | 4/8  Satisfactory | |
| Spergel J (2009)^35^ | Retrospective and prospective chart review | a (+1) | NA (0) | | a (+1) | NA (0) | | NA (0) | | b (+1) | | a (+1) | | | d (0) | | 4/8  Satisfactory | |
| Straumann A (2005)^31^ | Prospective case series | a (+1) | NA (0) | | a (+1) | NA (0) | | NA (0) | | b (+1) | | a (+1) | | | d (0) | | 4/8  Satisfactory | |
| Ukleja A (2014)^43^ | Retrospective chart review | a (+1) | NA (0) | | a (+1) | NA (0) | | NA (0) | | b (+1) | | a (+1) | | | d (0) | | 4/8  Satisfactory | |
| Weiler T (2014)^38^ | Retrospective chart review | a (+1) | NA (0) | | a (+1) | NA (0) | | NA (0) | | b (+1) | | a (+1) | | | d (0) | | 4/8  Satisfactory | |
| **Epidemiology studies**^†^ | | | | | | | | | | | | | | | | | | |
| Ally M (2015)^5^ | Administrative data review | a (+1) | NA (0) | | a (+1) | NA (0) | | NA (0) | | b (+1) | | NA (0) | | | NA (0) | | | NA |
| Arias A (2013)^19^ | Retrospective database review | a (+1) | NA (0) | | a (+1) | NA (0) | | NA (0) | | b (+1) | | NA (0) | | | NA (0) | | | NA |
| Cherian S (2006)^24^ | Retrospective audit | a (+1) | NA (0) | | a (+1) | NA (0) | | NA (0) | | b (+1) | | NA (0) | | | NA (0) | | | NA |
| Cohen M (2012)^23^ | Retrospective cohort study | a (+1) | NA (0) | | a (+1) | NA (0) | | NA (0) | | b (+1) | | NA (0) | | | NA (0) | | | NA |
| Dalby K (2010)^15^ | Prospective study | a (+1) | NA (0) | | a (+1) | NA (0) | | NA (0) | | b (+1) | | NA (0) | | | NA (0) | | | NA |
| Dellon E (2014)^6^ | Administrative data | a (+1) | NA (0) | | a (+1) | NA (0) | | NA (0) | | b (+1) | | NA (0) | | | NA (0) | | | NA |
| Dellon E (2015)^14^ | Retrospective registry review | a (+1) | NA (0) | | a (+1) | NA (0) | | NA (0) | | b (+1) | | NA (0) | | | NA (0) | | | NA |
| Gill R (2007)^7^ | Retrospective chart review | a (+1) | NA (0) | | a (+1) | NA (0) | | NA (0) | | b (+1) | | NA (0) | | | NA (0) | | | NA |
| Giriens B (2015)^20^ | Retrospective chart review | a (+1) | NA (0) | | a (+1) | NA (0) | | NA (0) | | b (+1) | | NA (0) | | | NA (0) | | | NA |
| Homan M (2015)^18^ | Retrospective chart review | a (+1) | NA (0) | | a (+1) | NA (0) | | NA (0) | | b (+1) | | NA (0) | | | NA (0) | | | NA |
| Hruz P (2011)^21^ and Straumann (2005)^22^ | Prospective database review | a (+1) | NA (0) | | a (+1) | NA (0) | | NA (0) | | b (+1) | | NA (0) | | | NA (0) | | | NA |
| Kim S (2015)^8^ | Retrospective database review | a (+1) | NA (0) | | a (+1) | NA (0) | | NA (0) | | b (+1) | | NA (0) | | | NA (0) | | | NA |
| Noel R (2004)^9^ | Retrospective population based demographic study | a (+1) | NA (0) | | a (+1) | NA (0) | | NA (0) | | b (+1) | | NA (0) | | | NA (0) | | | NA |
| O'Donnell S (2011)^16^ | Retrospective review of histopathology database | a (+1) | NA (0) | | a (+1) | NA (0) | | NA (0) | | b (+1) | | NA (0) | | | NA (0) | | | NA |
| Stewart M (2013)^11^ and Syed (2012)^12^ | Retrospective review | a (+1) | NA (0) | | a (+1) | NA (0) | | NA (0) | | b (+1) | | NA (0) | | | NA (0) | | | NA |
|  | **Case-control studies** | **Selection** | | | | | | **Comparability** | | **Exposure** | | | | | | | | **Total score** |
|  |  | Is the case definition adequate?  a) yes, with independent validation *****  b) yes, e.g., record linkage or based on self-reports  c) no description | Representativeness of the cases  a) consecutive or obviously representative series of cases *****  b) potential for selection biases or not stated | | Selection of Controls  a) community controls *****  b) hospital controls  c) no description | Definition of Controls  a) no history of disease (endpoint) *****  b) no description of source | | Comparability of cases and controls on the basis of the design or analysis  a) study controls for age and sex*  b) study controls for any additional factors* | | Exposure  Ascertainment of exposure  a) secure record (e.g. surgical records) *****  b) structured interview where blind to case/ control status*****  c) interview not blinded to case/ control status  d) written self report or medical record only  e) no description | | | Same method of ascertainment for cases and controls  a) yes *****  b) no | | | Non-Response rate  a) same rate for both groups *****  b) non respondents described  c) rate different and no designation | |  |
| **Natural history study** | | | | | | | | | | | | | | | | | | |
| Kinoshita Y (2013)^28^ | Questionnaire based survey | a (+1) | a (+1) | | b (0) | NA (0) | | NA (0) | | c (0) | | | a (+1) | | | NA (0) | | 3/8  Poor |
|  | **Cross-sectional studies** | **Selection** | | | | | | **Comparability** | | **Outcome** | | | | | | | | **Total score** |
|  |  | Representativeness of the sample:  a) Truly representative of the average in the target population. * b) Somewhat representative of the average in the target population. * c) Selected group of users.  d) No description of the sampling strategy | Sample size:  (> 50)  a) Justified and satisfactory. *  b) Not justified. | Non-respondents:  a) Comparability between respondents and non-respondents characteristics is established, and the response rate is satisfactory. *  b) The response rate is unsatisfactory, or the comparability between respondents and non-respondents is unsatisfactory. c) No description of the response rate or the characteristics of the responders and the non-responders | | Ascertainment of the exposure (risk factor):  a) Validated measurement tool. **  b) Non-validated measurement tool, but the tool is available or described.* c) No description of the measurement tool. | | The subjects in different outcome groups are comparable, based on the study design or analysis. Confounding factors are controlled.  a) The study controls for the most important factor (select one). *  b) The study control for any additional factor. * | | Assessment of the outcome  a) Independent blind assessment. **  b) Record linkage. **  c) Self report. *  d) No description. | Statistical test:  a) The statistical test used to analyze the data is clearly described and appropriate, and the measurement of the association is presented, including confidence intervals and the probability level (p value). *  b) The statistical test is not appropriate, not described or incomplete. | | | ---- | | | |  |
| **Natural history studies** | | | | | | | | | | | | | | | | | | |
| Castro Jimenez A (2014)^29^ | Prospective cross-sectional study | a (+1) | b (0) | A (+1) | | c (0) | | NA (0) | | b (+2) | b (0) | | | --- | | | | 4/10  Poor |
| Menard-Katcher P (2013)^40^ | Retrospective cross-sectional study | a (+1) | a (+1) | A (+1) | | a (+2) | | NA (0) | | b (+2) | a (+1) | | | --- | | | | 8/10  Good |
| Moawad F (2016)^34^ | Retrospective cross-sectional study | a (+1) | a (+1) | A (+1) | | b (+1) | | NA (0) | | b (+1) | a (+1) | | | --- | | | | 6/10  Satisfactory |
| **Epidemiology studies**^†^ | | | | | | | | | | | | | | | | | | |
| Van Rhijn B (2013)^17^ | Retrospective cross-sectional study of histo- and cytopathology database reports | a (+1) | NA (0) | A (+1) | | NA (0) | NA (0) | | b (+1) | | a (+1) | | | d (0) | | | | 4/8  Satisfactory |

NA, not applicable.

^†^The applicability of the Newcastle-Ottawa scale for epidemiology studies was limited. Mansoor E & Cooper G (2016)^13^ and Prasad G (2009)^10^ are covered under natural history studies.
